# The Spatial Distributions and Variations of Water Environmental Risk in Yinma River Basin, China

**DOI:** 10.3390/ijerph15030521

**Published:** 2018-03-15

**Authors:** Hui Di, Xingpeng Liu, Jiquan Zhang, Zhijun Tong, Meichen Ji

**Affiliations:** School of Environment, Northeast Normal University, Changchun 130024, China; dih717@nenu.edu.cn (H.D.); zhangjq022@nenu.edu.cn (J.Z.); jimc889@nenu.edu.cn (M.J.)

**Keywords:** Yinma River Basin, PSO-AHP method, water environmental risk, risk assessment

## Abstract

Water environmental risk is the probability of the occurrence of events caused by human activities or the interaction of human activities and natural processes that will damage a water environment. This study proposed a water environmental risk index (WERI) model to assess the water environmental risk in the Yinma River Basin based on hazards, exposure, vulnerability, and regional management ability indicators in a water environment. The data for each indicator were gathered from 2000, 2005, 2010, and 2015 to assess the spatial and temporal variations in water environmental risk using particle swarm optimization and the analytic hierarchy process (PSO-AHP) method. The results showed that the water environmental risk in the Yinma River Basin decreased from 2000 to 2015. The risk level of the water environment was high in Changchun, while the risk levels in Yitong and Yongji were low. The research methods provide information to support future decision making by the risk managers in the Yinma River Basin, which is in a high-risk water environment. Moreover, water environment managers could reduce the risks by adjusting the indicators that affect water environmental risks.

## 1. Introduction

Water environments refer to water bodies that directly or indirectly influence human life and developments surrounding a population, and these environments provide the basis for human activities [1]. With the acceleration of the urbanization process and the progressive development of industry and agriculture, water environments have been affected by human activities in different ways [2]. Pollutants in the water body emitted by human activities mainly include total phosphorus (TP), total nitrogen (TN) and Chemical Oxygen Demand (COD) [3], and these pollutants increase the risks to the water environment, which has become a severe problem for social and ecological systems. These increased risks have caused adverse effects on the health of residents such as increases in morbidity and mortality [4,5]. Some engineering projects can also cause water pollution, for example, hydroelectric projects are an important project source. The relationship between hydroelectric projects and the river environment in Ningxia Reach of the Yellow River in China was analyzed, where the results showed that it was necessary to coordinate the exploitation of water resources and the construction of the hydropower project with the maintenance of the health of the river system [6]. Additionally, due to the lack of reasonable planning, water pollution events such as industrial water pollution and heavy metal pollution have frequently occurred in several watersheds [7,8]. The question of how to prevent and avoid the occurrence of water pollution events has become an urgent problem that needs to be solved; water environmental risk assessments can reflect the level of water environmental risk, and then, corresponding measures could be taken to reduce the risks in high-risk areas.

Recent research on water environmental risks can be classified into two categories: (1) Ecological risk assessments. An ecological risk assessment is mainly carried out by selecting typical pollutants and conducting sampling to determine the distribution of pollutants in a river; then, the ecological risk is calculated using an ecological risk assessment model such as food web-based models, ecosystem-based models, and socio-ecological models [9,10,11]; (2) Human health risk assessments. Water pollution affects human health via direct drinking, crop irrigation, livestock farming, the fishery industry, etc. Human health risk assessments provide a method to measure the risks to human health caused by natural changes to and the pollution of a water environment. At present, studies on heavy metal and organic pollutant health risk assessments have been implemented in areas such as the Maocun River, Dongjiang River, Poyang Lake, and Huaihe River in China [12,13,14,15]. In addition, geographic information system (GIS) is widely used in the field of water environmental risk assessments [16,17], and integrates data collection, spatial analysis, and decision-making processes into a common information flow, which significantly improves work efficiency and provides technical support for solving water environmental problems and ensuring sustainable development.

The risk factors of the water environment in a basin include risk sources, natural factors, and socioeconomic factors. The sources that cause water environmental risks include mainly agricultural pollution, industrial pollution, and anthropogenic pollution. The main pollutants of agricultural pollution include nutrients such as nitrogen and phosphorus, organic pesticides, chemical fertilizers, and other organic or inorganic pollutants. These pollutants cause environmental pollution through surface runoff and underground leakage, which can be transported into residential soil and drinking water sources, so agricultural activities increase the environmental and human health risks [18,19]. Organic pollutants, inorganic pollutants, and heavy metals account for a large proportion of industrial pollution, and these pollutants originate from printing and dyeing, coal mining, leather manufacturing, metal manufacturing, etc. Anthropogenic pollution mainly refers to the discharge of domestic sewage. Climate change can cause the deterioration of water quality, which leads to a series of serious water environmental risks. For example, rainfall and atmospheric sedimentation will result in the pollution of persistent organic pollutants such as nitrogen and phosphorus in surface water. In addition, there is also a correlation between economic development and water quality, and the research has fully considered the impacts of economic indicators, environmental water quality indicators, social indicators, and natural environmental indicators on economic development and water basin environmental risks [20,21]. Due to the extensive sources and complicated components, water environmental risks are difficult to control [22]; thus, water environmental risk assessments are essential for effectively controlling water environmental risks.

The problem regarding water environmental risks has aroused wide attention, how to assess the water environmental risks has become a hot topic, and the selection of evaluation indicators is the key to water environmental risks assessment. In this study, several indicators such as economic indicators, environmental water quality indicators, social indicators, and natural environmental indicators were selected to assess water environmental risks. The purpose of this study was to analyze the water environmental risk of the Yinma River Basin based on four water environmental risk factors: hazards, exposure, vulnerability, and regional management ability, and the existing assessment indicators were reclassified according to the definitions of the four factors. Based on the existing indicators, this study added soil erosion as a natural environmental indicator to reflect hazards, and the standard-reaching rate of the industrial wastewater and domestic sewage, and rate of treated domestic garbage were added as water environmental quality indicators to reflect the regional management ability. A risk index model was proposed to analyze the water environmental risk in the Yinma River Basin, and then, water environmental risk maps in 2000, 2005, 2010, and 2015 were drawn to show the variations in water environmental risk in the Yinma River Basin.

## 2. Materials and Methods

### 2.1. Study Area 

**Figure 1:**
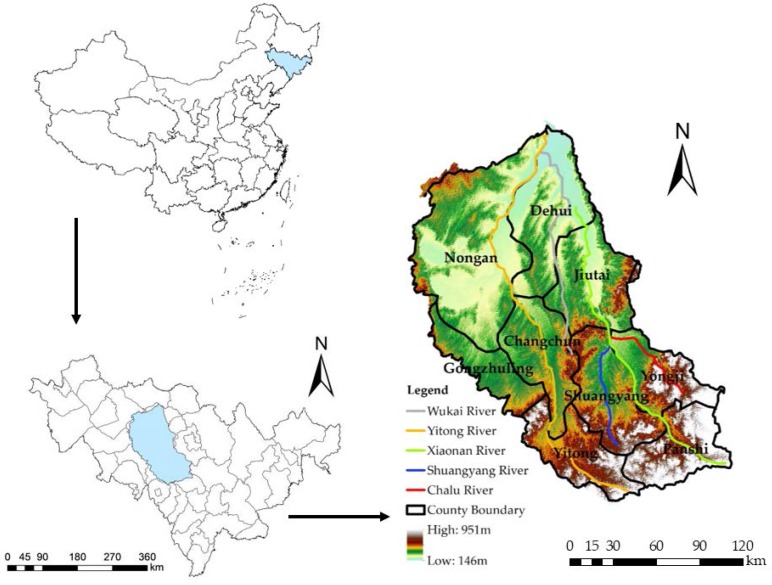
Location of the Yinma River Basin, China.

The Yinma River Basin is located in the center of Jilin Province, China and is an important branch of the Songhua River fluvial system (Figure 1), which originates from the Hulan Ridge of Panshi City, Jilin Province, and flows into the Songhua River in the Kaosan town of Nongan. The Yinma River flows through six counties including Panshi, Shuangyang, Yongji, Jiutai, Dehui, and Nongan. The length is 386.8 km, and the watershed area is approximately 17,400 km^2^. The Yinma River Basin lies within a typical continental monsoon climate. The mean annual temperature of the area is 5.3 °C. The mean annual precipitation ranges from 370 to 668 mm, and mean annual evaporation is 1438.4 mm. The terrain is high in the southeast and low in the northwest. The mean total annual water resources are 2.472 billion m^3^, and the per capita water resources are 378 m^3^, which accounts for 18% of the total in China.

The study area included the entire area of Changchun city and portions of Jilin city and Siping city. The Yinma River flows through the core area of northeast black soil, which is the center of the economic district and is a production zone for rice, corn, and other crops in Jilin Province. The area is also the main grain base and the northeast industrial base in China. Statistics from 2016 indicate that the total population of the area was 8.9 million, the urbanization rate was 68.4%, and the annual GDP was 400 billion yuan, accounting for 40% of the province total and 21.4% of the entire Songhua River Basin. The pollution sources of the Yinma River mainly include agricultural, industrial, and residential activities such as the use of chemical fertilizers and pesticides, the discharge of industrial wastewater, and domestic sewage. Due to the improper protection and utilization of water resources, risks to the water environment have gradually increased in this area.

### 2.2. Data Collection

The data used in this study were (1) meteorological data. The meteorological data for this study were downloaded from the China meteorological data sharing service system. The daily rainfall data for 2000, 2005, 2010, and 2015 were collected from a total of 19 meteorological stations in the Yinma River Basin. These data were used to calculate soil erosion; (2) Socio-economic data. These data were obtained from the Jilin Statistical Yearbooks for 2001, 2006, 2011, and 2016. These data included information on chemical fertilizer and pesticide application amounts, industrial wastewater emissions, domestic sewage emissions, standard-reaching rates of the industrial wastewater and domestic sewage, and the rate of untreated domestic garbage; (3) Water resources data. These data were obtained from the water resources bulletins of Jilin Province for 2000, 2005, 2010, and 2015 and included the per-capita water consumption and per-10,000-yuan-GDP water consumption; (4) Basic geographical data. These data included an administrative map of the study area and digital elevation model (DEM) data. The elevation and slope were obtained from the DEM. The DEM, soil and land use data were downloaded from the Data Center for Resources and Environmental Sciences, Chinese Academy of Sciences (RESDC) (All of these data were included in the Supplementary Materials).

### 2.3. Methods

#### 2.3.1. Establishment of Water Environmental Risk Assessment Framework

**Figure 2:**
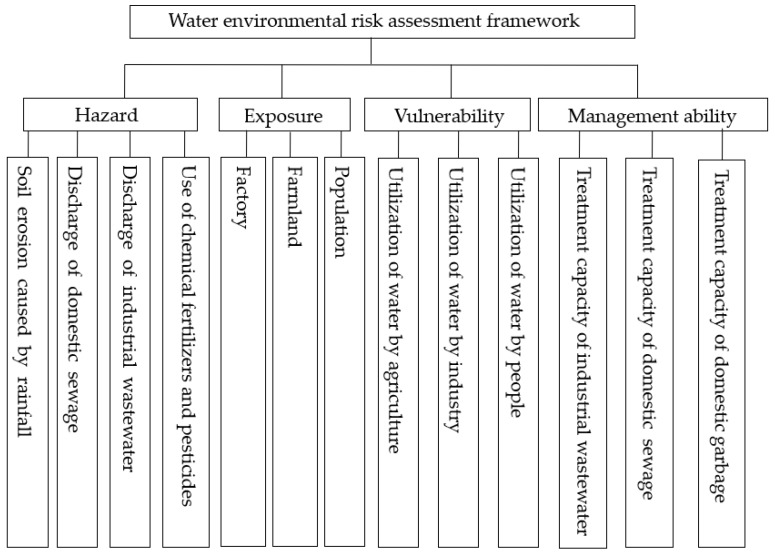
The water environmental risk assessment framework.

In this study, the water environmental risk was considered a comprehensive effect of four factors: hazards, exposure, vulnerability, and regional management ability in the water environment. A water environmental risk assessment framework was established according to the definitions of the four factors. Hazards describe the external danger of water pollution and are affected by soil erosion, domestic sewage, and industrial wastewater discharge, as well as chemical fertilizer and pesticide usage. Exposure describes the important objects that are affected by water pollution, farmland area, number of factories, and population scale. Vulnerability describes the response degrees of different objects to water environmental hazards and is affected by the utilization of water by agriculture, industry, and people. Regional management ability describes the water pollution management level and is affected by the industrial wastewater, domestic sewage, and domestic garbage treatment levels. Figure 2 shows the water environmental risk assessment framework.

#### 2.3.2. Particle Swarm Optimization and the Analytic Hierarchy Process (PSO-AHP) Model 

The PSO-AHP method was presented in 2011 to select the best suppliers and address the issue of product part changes [23]; it has also been used to determine the weights of indicators in some studies [24]. The analytic hierarchy process (AHP) is an analytic technique for multiobjective decisions combined with qualitative and quantitative analysis, and determines the weights of factors by using the multifactor classification method. Particle swarm optimization (PSO) is a global optimization algorithm that originated from research on the predatory behaviors of birds. Similar to a genetic algorithm, a PSO is an algorithm based on iterations, and the basic idea is that a group (particle swarm) is randomly initialized, and then, the velocity and position of each individual (particle) in the group is revised through an iterative process until the algorithm ends. To maintain the original information from the decision makers and ensure consistency of the judgment matrix, PSO was applied to the AHP; then, a PSO-AHP model was constructed to determine the weights of the relative indicators in this study. The operation process was as follows:

(1) Hierarchical structure of the comprehensive evaluation system.

The comprehensive evaluation system had three layers recorded as A, B, and C from the top to the bottom of the index layer. Layer A represented the overall goal of the system evaluation and has only one element, while the number of elements in layers B and C were recorded as *n_b_* and *n_c_*, respectively.

(2) Structure judgment matrix.

For the elements in layers B and C, a pairwise comparison was conducted with the elements from the above layer, where a 1–9 scale method is usually used to describe the relative importance of each factor. The judgment matrix of layer B: *A_K_*= (*a_ij_*)*_nb×nb_*. Element *a_ij_* represents the relative importance of factor *B_i_* to factor *B_j_* from the perspective of layer A. The judgment matrix of layer C: *B_K_*= {$b_{ij}^{k}$*|i*, *j* = 1-*n_c_*; *k* = 1-*n_b_*}*_nc×nc_.* A comparison was made between the criteria and the applicable alternatives using the assessment scale shown in Table 1, and comparison matrices were formed [25,26].

(3) Establish the weight optimization model.

The optimization of the weight value corresponding to the judgment matrix *A_K_* = (*a_ij_*)*_nb×nb_* was illustrated as an example; then, the objective function was defined:

(1) $$\mathrm{MinCIF}\left(n_{b}\right)=\overset{n_{b}}{\underset{i=1}{\sum }}\left|\overset{n_{b}}{\underset{k=1}{\sum }}\left(a_{ik}w_{k}\right)-n_{b}w_{i}\right|/n_{b}$$

(2) $$s.t.\;w_{k}>0,\;k=1-n_{b}$$

(3) $$\overset{n_{b}}{\underset{k=1}{\sum }}w_{k}=1$$

where $\mathrm{CIF}\left(n_{b}\right)$ represents the consistency index function; *n_b_* represents the number of elements in layer B; and *w_k_* represents the single ranking weight of each element in layer B.

(4) Solve the weight optimization model using the PSO algorithm.

The parameters of the PSO algorithm included the number of particles *n*, the maximum number of iterations *N*, the two learning factors *c*_1_ and *c*_2_, and the range of variation in the inertia coefficient *v_in_*, [−*v_max_*, *v_max_*]. In this study, *n* = 10, *N* = 3000, *c*_1_ = *c*_2_ = 2, and the range of variation in *v_in_* was [−0.3, 0.3]. The steps of the PSO algorithm are as follows:

Step 1 Generate the initial solution of particles

Generate a random number in the solution space (0,1) and normalize the generated random number to make it a feasible solution.

Step 2 Calculate the fitness of the initial particle

Import the feasible solution into the objective function and select the best global particle.

Step 3 Update the particle iterations

The individual optimal value is the particle in the first iteration of the initial particle; in the subsequent iterations, the optimal value for the individual is the best point identified when the solution space moved.

Step 4 Determine whether the updated particles meet the constraint conditions

If the constraint conditions are not met, the particles should be normalized.

Step 5 Calculate the fitness levels of the updated particles 

The optimal particle positions and the optimal global positions are compared and selected.

Step 6 Judge whether the optimal solution meets the iterative termination condition

If the condition satisfies the iterative termination condition, the iteration stops; then, the optimal solution obtained from the model is outputted and move to step 7; if the condition is not satisfied, return to step 3 and resume the process.

Step 7 Calculate the consistency ratio value corresponding to the judgment matrix

If it does not meet the consistency requirements, adjust the judgment matrix by the maximum direction improvement method and the interval number improvement method, then return to step 1 and re-execute the process.

(5) Output the global optimal position, the corresponding weight value, and the consistency index function value.

#### 2.3.3. Water Environmental Risk Index (WERI) Model

In this study, a water environmental hazard was defined as the degree of danger that may be caused by a water pollution incident, which is mainly decided by the strengths of the events. Water environmental exposure was defined as the people, economy, ecosystem, or other real estate that may be affected by water pollution. Water environmental vulnerability was defined as the degree of the responses by people, the social economy, and the ecological environment. Water environmental regional management ability was defined as the degree of water pollution management by a local government. According to the formation mechanisms of water environmental risks, the WERI model was established by integrating the four factors and 13 indicators:

WERI = H^WH^ × E^WE^ × V^WV^ × (1 − G) ^WG^ (4)

(5) $$H=\overset{n}{\underset{i=1}{\sum }}X_{hi}\times W_{hi}$$

(6) $$E=\overset{n}{\underset{i=1}{\sum }}X_{ei}\times W_{ei}$$

(7) $$V=\overset{n}{\underset{i=1}{\sum }}X_{vi}\times W_{vi}$$

(8) $$G=\overset{n}{\underset{i=1}{\sum }}X_{gi}\times W_{gi}$$

where WERI is the water environmental risk index; *H*, *E*, *V*, and *G* are the water environmental hazard, exposure, vulnerability and regional management ability index, respectively; *W* is weight value of each evaluation indicator; and *X* is the quantitative value of each evaluation indicator.

## 3. Results

### 3.1. Weights of Evaluation Indicators Calculated by the PSO-AHP Model

According to the process of water environmental risk formation, the evaluation indicators were selected from the perspectives of hazards, exposure, vulnerability, and regional management ability, and the evaluation indicators were layered based on the water environmental risk assessment framework; the results are shown in Table 2.

A judgment matrix was constructed by the experts in the field of water environmental risk assessments according to the layers in Table 2, and the results are shown as follows:

$$\begin{array}{c} A=\left[\begin{array}{cccc} 1 & 1/2 & 1/3 & 2\\ 2 & 1 & 1/2 & 3\\ 3 & 2 & 1 & 4\\ 1/2 & 1/3 & 1/4 & 1 \end{array}\right]\;B_{1}=\left[\begin{array}{cccc} 1 & 1/7 & 1/5 & 1/5\\ 7 & 1 & 1/2 & 3\\ 5 & 2 & 1 & 1/3\\ 5 & 1/2 & 3 & 1 \end{array}\right]\\ B_{2}=\left[\begin{array}{ccc} 1 & 1/7 & 1/3\\ 7 & 1 & 1\\ 3 & 1 & 1 \end{array}\right]\;B_{3}=\left[\begin{array}{ccc} 1 & 1 & 3\\ 1 & 1 & 3\\ 1/3 & 1/3 & 3 \end{array}\right]\;B_{4}=\left[\begin{array}{ccc} 1 & 1 & 1/2\\ 1 & 1 & 2\\ 2 & 1/2 & 1 \end{array}\right] \end{array}$$

Referring to the process used to solve the PSO-AHP model, the value of the consistency index function was obtained by programming in MATLAB2016b software using a global solver, and the weight value for the matrix and the value of the consistency index function are shown in Table 3 (CIF < 0.1 means the judgment matrix has a good consistency).

### 3.2. Analysis of Water Environmental Hazards

**Figure 3:**
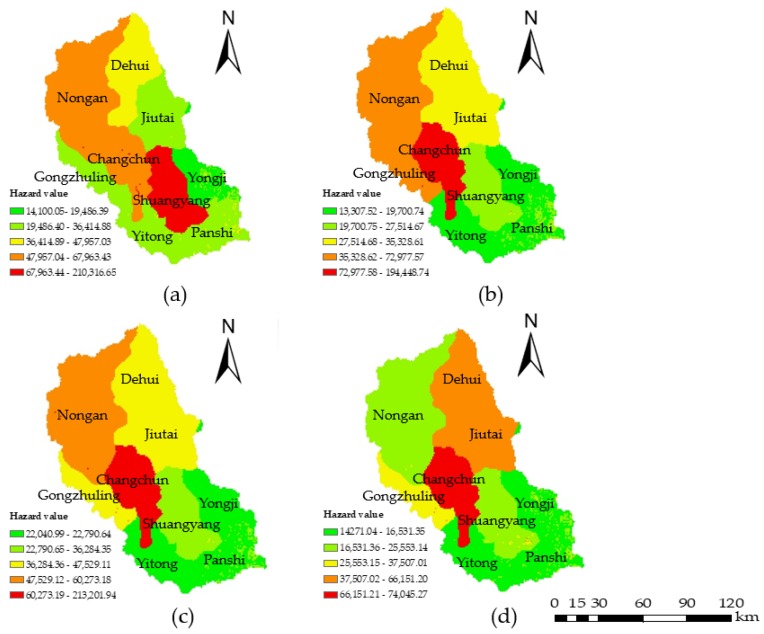
Water environmental hazard maps for (**a**) 2000; (**b**) 2005; (**c**) 2010; and (**d**) 2015. The color scale from green to red represents slightly hazardous, lightly hazardous, moderately hazardous, highly hazardous, and extremely hazardous, respectively.

The water environmental hazard analysis mainly considered four indicators: soil erosion emissions, chemical fertilizer and pesticide application amounts, industrial wastewater emissions, and domestic sewage emissions. Data from 2000, 2005, 2010, and 2015 were selected, and the four hazard indicators were projected on the distribution map. The hazard values were calculated by Equation (5) using the grid calculator (Figure 3) in ArcGIS (version 10.2; ESRI, Redlands, CA, USA). The classification was based on the natural discontinuity classification method (the exposure, vulnerability, regional management ability, and risk values were similarly calculated). The results showed that from 2000 to 2015, the water environmental hazards gradually decreased throughout the area, and the high hazard areas were mainly concentrated in the central and north-central regions such as Changchun, Nong’an, Dehui, and Jiutai city, which was due to the use of large amounts of chemical fertilizers and pesticides, industrial wastewater emissions, and domestic sewage emissions. As the capital city of Jilin Province, Changchun has better development than the other cities, so the emissions of industrial wastewater and domestic sewage are higher; this is the reason why the hazard value in Changchun is always high. The hazard level was high in 2000 in this area, as large amounts of soil erosion emissions were caused by the heavy rainfall that year. The spatial distributions were similar in 2005 and 2010 due to the inconspicuous variation of each indicator; however, with the continuous development of society and the economy, more attention has been paid to the problem of water pollution. Thus, corresponding measures were taken to control the use of chemical fertilizers and the emissions of industrial wastewater, which led to the reduction in hazards in 2015.

### 3.3. Analysis of Water Environmental Exposure

**Figure 4:**
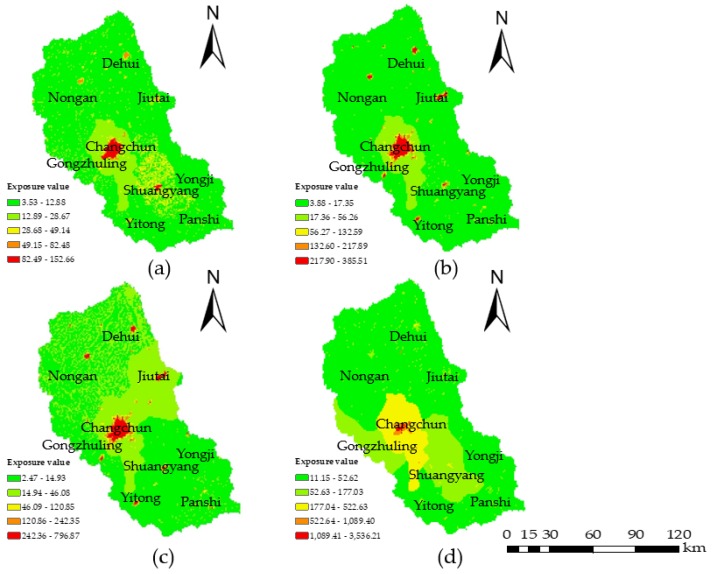
Water environmental exposure maps for (**a**) 2000; (**b**) 2005; (**c**) 2010; and (**d**) 2015. The color scale from green to red represents slightly exposed, lightly exposed, moderately exposed, highly exposed, and extremely exposed, respectively.

From the perspective of the extent of exposure to agriculture, residents and industry, the farmland area, population density, and number of industrial enterprises were selected to describe the water environmental exposure. Data from 2000, 2005, 2010, and 2015 were selected, and the three exposure indicators were projected on the distribution map; the exposure value was calculated by Equation (6) using the grid calculator (Figure 4). The temporal and spatial distributions of the exposure levels from 2000 to 2015 indicated that the water environmental exposure gradually increased. Due to the low population density and low rates of industrial and agricultural developments, the exposure levels of most of the regions in the study area, except for the central area, were slight or low. Since 2010, sustainable development has been implemented to improve agriculture, forestry, and animal husbandry; the cultivated land area has expanded; and the population has continuously grown, which resulted in the increase in exposure.

### 3.4. Analysis of Water Environmental Vulnerability

**Figure 5:**
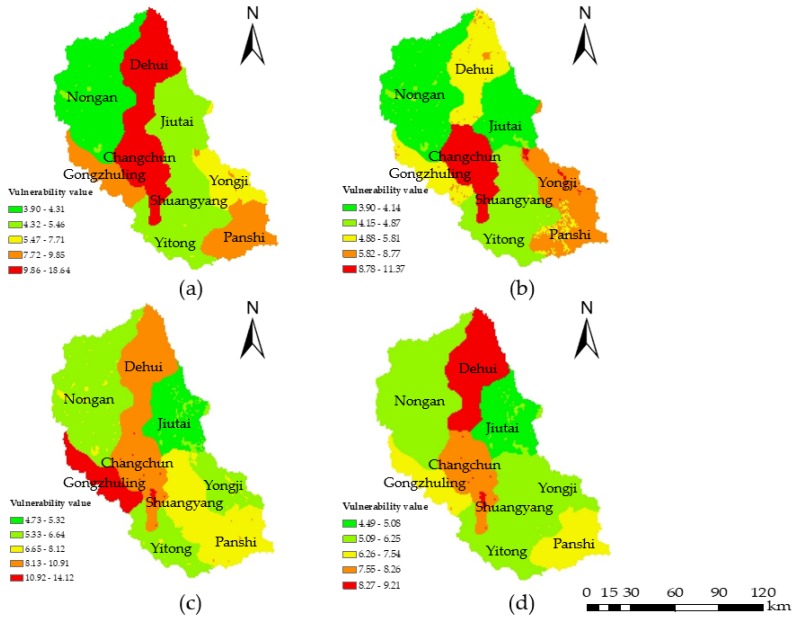
Water environmental vulnerability maps of (**a**) 2000; (**b**) 2005; (**c**) 2010; and (**d**) 2015. The color scale from green to red represents slightly vulnerable, lightly vulnerable, moderately vulnerable, highly vulnerable, and extremely vulnerable, respectively.

The vulnerability was analyzed based on the degree of water resource utilization by regional social characteristics. The vulnerability was described by three indicators: irrigation area, per-capita water consumption, and per-10,000-yuan-GDP water consumption. Data from 2000, 2005, 2010, and 2015 were selected, and the three vulnerability indicators were projected on the distribution map. The vulnerability values were calculated by Equation (7) using the grid calculator (Figure 5). The results showed that most of the extremely, highly, and moderately vulnerable areas were distributed in the north-central and southeast regions. The reason for this situation is that irrigated lands are widely distributed in the north and southeast of the study area, and the urbanization zones with high population densities and industrial outputs are concentrated in the central regions. These vulnerable zones are mainly disturbed near the river. The high vulnerability in the city of Panshi is due to the headstream of the Yinma River, and Dehui is located near the outlet of the Yinma River that flows into the Songhua River. In addition, there were many scattered points distributed throughout the basin that coincided with the distribution of rural areas. The rural areas were heavily disturbed by the utilization of fertilizers; therefore, these areas were more vulnerable than the surrounding areas. This pattern explains why the points of serious vulnerability levels were present in slight vulnerability level extents. As a whole, the vulnerability of the basin decreased from 2000 to 2015.

### 3.5. Analysis of Water Environmental Regional Management Ability 

**Figure 6:**
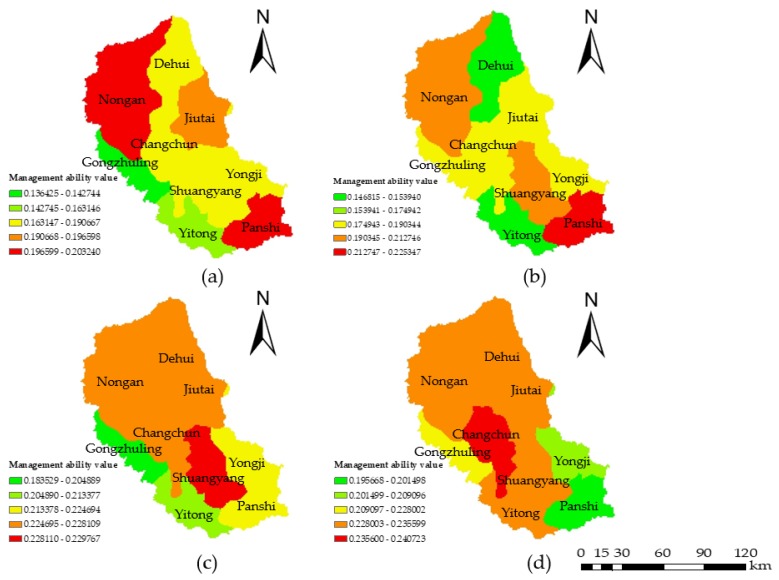
Water environmental regional management ability maps of (**a**) 2000; (**b**) 2005; (**c**) 2010; and (**d**) 2015. The color scale from green to red represents slightly managed, lightly managed, moderately managed, heavily managed, and extremely managed, respectively.

From the perspective of the control of water pollution, the water environmental management ability was used to analyze the ability of a region to manage industrial wastewater, domestic sewage, and domestic garbage. Therefore, three indicators including the standard-reaching rate of industrial wastewater, standard-reaching rate of domestic sewage, and rate of untreated domestic garbage were analyzed. Data from 2000, 2005, 2010, and 2015 were selected, and the three regional management ability indicators were projected on the distribution map. The regional management ability value was calculated by Equation (8) using the grid calculator (Figure 6). The results showed that the regional water environmental management ability gradually increased from 2000 to 2015, which indicated that the government paid more attention to the water pollution problem, and the treatment of industrial wastewater achieved satisfactory results. However, the effectiveness of domestic sewage and garbage treatments should be strengthened in the high hazard areas.

### 3.6. Analysis of Water Environmental Risk

**Figure 7:**
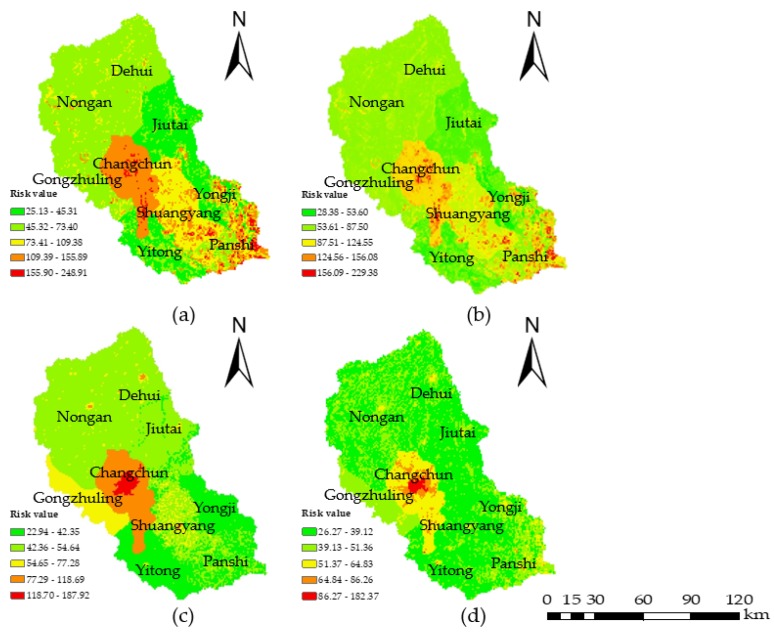
Water environmental risk maps of (**a**) 2000; (**b**) 2005; (**c**) 2010; and (**d**) 2015. The color scale from green to red represents slightly risky, lightly risky, moderately risky, highly risky, and extremely risky, respectively.

According to the conceptual framework and the water environmental risk assessment model, the water environmental risk index (WERI) was calculated using the raster calculator in ArcGIS (version 10.2; ESRI, Redlands, CA, USA) according to Equation (4). Figure 7 shows that the overall water environmental risk decreased from 2000 to 2015. The reason for this variation tendency was that people paid more attention to the problem of water pollution and corresponding measures were taken to reduce water pollution. From the view of different administrative areas, Changchun consistently had a high level of risk, while Yitong and Yongji always had low levels of risk, which was related to the local social and economic development. Combined with the analysis of the weight of each indicator, the hazard and exposure indicators accounted for larger proportions; therefore, the water environmental risk was mainly determined by the variations in hazards and exposure. Equation (4) shows that the water environment risk is proportional to the hazard, exposure and vulnerability, while it is inversely proportional to the ability of a region to manage the water environment, which is why the water environmental risk decreased from 2000 to 2015.

## 4. Discussion

The results showed that from 2000 to 2015, the water environmental risk gradually decreased in the Yinma River Basin. This decrease occurred because Jilin Province strengthened the construction of the urban drainage network and sewage treatment facilities, and a large number of pollution sources were incorporated into the municipal sewage pipe network, which reduced water pollution. Considering the situation of domestic and foreign research, this study proposed a water environmental risk index model to analyze the water environmental risk based on four factors, and this study provides a reference for the establishment of an early risk warning system for the water environment; thus, correlated research on early warnings for water environmental risk should be carried out from the perspectives of these four factors.

The allocation of weight across the four factors that affect water environmental risk indicated that hazards accounted for a large proportion, and the hazard indicators reflected the condition of water pollution. Wu [27] selected seven monitoring sections in the reservoirs of the Yinma River and analyzed the changes in ammonia nitrogen and the chemical oxygen demand in each section from 2003 to 2011, and the results showed that water pollution in the Yinma River has decreased. Studies have shown that with the increase in runoff, the dilution and self-purification abilities of water will be enhanced; then, the degree of water pollution will relatively decrease [28,29,30]. The observed and statistical data from the Dehui Hydrological Station in the Yinma River Basin indicated that runoff gradually increased from 2000 to 2015 in the study area; therefore, the degree of water pollution gradually weakened, and these results were consistent with the decrease in the water environmental risk from 2000 to 2015.

This study was a large-scale analysis of social and economic indicators and did not consider the types of pollutants in the water bodies or their potential effects on human health. Water environmental risk includes several risk factors and complex development processes; therefore, future studies on water environmental risk should not only consider the risk formation mechanisms, the risk transmission modes, the degree of sensitivity of the risk receptor, and the four factors mentioned in this study, but also analyze the damage caused to the social economy and human health. Then, corresponding risk prevention measures should be established to effectively control the risks to the water environment.

## 5. Conclusions

To assess the water environmental risk of the Yinma River Basin, the hazards, exposure, vulnerability, and regional management ability were defined to develop a water environmental risk framework and indicator system, and the WERI model was proposed based on the PSO-AHP method. With this model, the spatial-temporal distribution characteristics in 2000, 2005, 2010, and 2015 were analyzed, and the conclusions were as follows: The overall risk of the water environment demonstrated a decreasing trend in the study area. In addition, a majority of the extremely risky, highly risky, and moderately risky areas were distributed in the central region and some scattered areas in the north region of the study area where disturbances from economic development were severe. The slightly risky and lightly risky areas were in other regions, especially in the mountainous southeast region, given that mountains act as natural barriers to human activities; therefore, the wastewater emissions were low in this region. Based on the distribution of water environmental risk in the different administrative regions, the low and slight water environmental risk levels were common in Yitong and Yongji County, while the high water environmental risk level was in Changchun city

## Figures and Tables

**Table 1:** The 1–9 scale for the pairwise comparisons in the Analytic Hierarchy Process (AHP).

Importance Intensity	Definition
1	Equal importance
3	Moderate importance of one over another
5	Strong importance of one over another
7	Very strong importance of one over another
9	Extreme importance of one over another
2, 4, 6, 8	Intermediate values
Reciprocals	Reciprocals for inverse comparison

**Table 2:** The layers of the evaluation indicator system.

Target Layer	Factor Layer	Indicator Layer
	Hazard (B_1_)	Soil erosion emission (C_1_) Application amounts of chemical fertilizer and pesticide (C_2_) Industrial wastewater emission (C_3_) Domestic sewage emission (C_4_) Population density (C_5_)
	Exposure (B_2_)	
Water environmental risk (A)		Farmland area (C_6_)
	Vulnerability (B_3_)	Number of factories (C_7_)
		Irrigation area (C_8_)
	Regional management ability (B_4_)	Per-capita water consumption (C_9_) Per-10,000-yuan-GDP water consumption (C_10_) Standard-reaching rate of industrial wastewater (C_11_)
		Standard-reaching rate of domestic sewage (C_12_)
		Rate of treated domestic garbage (C_13_)

**Table 3:** Calculation results of the judgment matrix based on the PSO-AHP model.

Judgment Matrix	W_1_	W_2_	W_3_	W_4_	CIF
A	0.378	0.253	0.172	0.197	0.00180
B_1_	0.275	0.227	0.229	0.269	0.00178
B_2_	0.091	0.515	0.394		0.01210
B_3_	0.342	0.365	0.293		0.00217
B_4_	0.361	0.329	0.310		0.00195

## Supplementary Materials

The following are available online at http://www.mdpi.com/1660-4601/15/3/521/s1.
