# Supplementary figures and images for: The Spatial Distributions and Variations of Water Environmental Risk in Yinma River Basin, China
Source: Int J Environ Res Public Health. 2018 Mar 15;15(3):521. doi: 10.3390/ijerph15030521 (PMC5877066; doi:10.3390/ijerph15030521)

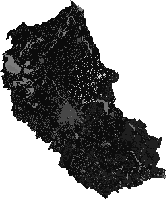

Supplement: Supplementary file 1 [file ijerph-15-00521-s001.zip › supplementary files/Landuse 2000/Landuse 2000.tif]

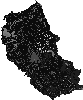

Supplement: Supplementary file 1 [file ijerph-15-00521-s001.zip › supplementary files/Landuse 2000/Landuse 2000.tif.ovr]

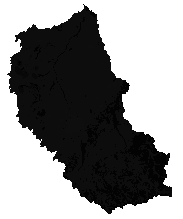

Supplement: Supplementary file 1 [file ijerph-15-00521-s001.zip › supplementary files/Landuse 2005/Landuse 2005.tif]

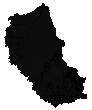

Supplement: Supplementary file 1 [file ijerph-15-00521-s001.zip › supplementary files/Landuse 2005/Landuse 2005.tif.ovr]

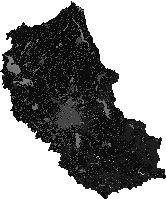

Supplement: Supplementary file 1 [file ijerph-15-00521-s001.zip › supplementary files/Landuse 2010/Landuse 2010.tif]

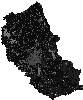

Supplement: Supplementary file 1 [file ijerph-15-00521-s001.zip › supplementary files/Landuse 2010/Landuse 2010.tif.ovr]

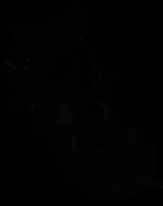

Supplement: Supplementary file 1 [file ijerph-15-00521-s001.zip › supplementary files/Landuse 2015/LandUse 2015.tif]

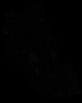

Supplement: Supplementary file 1 [file ijerph-15-00521-s001.zip › supplementary files/Landuse 2015/LandUse 2015.tif.ovr]
